# Waveguide-based augmented reality displays: a highlight

**DOI:** 10.1038/s41377-023-01371-4

**Published:** 2024-01-18

**Authors:** Jannick P. Rolland, Jeremy Goodsell

**Affiliations:** 1 Institute of Optics, University of Rochester, 480 Intercampus Drive, Rochester, NY 14627 USA; 2 Center for Freeform Optics, University of Rochester, Rochester, NY 14627 USA; 3 Center for Visual Science, University of Rochester, Rochester, NY 14627 USA

**Keywords:** Displays, Imaging and sensing

## Abstract

Augmented reality (AR), which emerged in the 1960s, remains a focal point of interest given its capacity to overlay the real world with digitally presented information through optical combiners. The prevalent combiner, commonly known as the waveguide in the AR literature, is prized for its compact design and generous eyebox—essential elements in human-centric technology. Nonetheless, these combiners encounter unique challenges in meeting various other requirements of the human visual system. This paper highlights a recent review of technological advancements and presents a forward-looking perspective on the future of AR technology.

Augmented reality (AR), tracing its roots to the 1960s, continues to capture broad attention for its capacity to enhance our visual reality. It is increasingly becoming an integral tool in various cutting-edge fields such as education, the arts, manufacturing, and medicine. From Ivan Sutherland’s groundbreaking Sword of Damocles AR display in 1968, head-worn AR displays have significantly reduced in size, now fitting into an eyeglass form factor^1,2^. The optical combiner, a crucial component of any visual AR display, enables simultaneous viewing of the real world and relaying digital information from the display.

Optical combiners are available in various form factors and architectures, with the waveguide combiner standing out as the most popular choice given its compact design and generous eyebox^3^. After coupling light into the waveguide, the waveguide combiner leverages total internal reflection (TIR) to guide the light into the waveguide, as depicted in Fig. 1. When the light encounters the expander region, each ray is replicated as it goes through a series of interactions with mirrors or gratings for the geometric or diffractive waveguides, respectively. The replication expands the eyebox along one dimension. The replicated and redirected light then interacts with the out-coupler, further expanding the light along the orthogonal direction and directing it toward the user’s eye. These mechanisms enlarge the eyebox without compromising the full field-of-view (FOV), ultimately increasing the system’s etendue at the expense of displayed brightness.

**Fig. 1:**
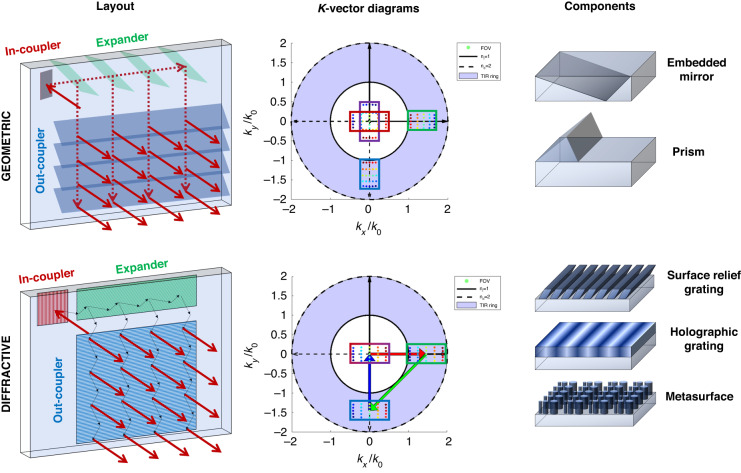
**Illustration of waveguide combiners.** The top row shows a geometric waveguide layout, the corresponding *k*-vector diagram, and examples of geometric waveguide components. The bottom row shows a diffractive waveguide layout, the corresponding *k*-vector diagram, and examples of diffractive waveguide components. In the *k*-vector diagrams, the FOV is shown by the rainbow-colored dots, and the incident FOV on the in-coupler, expander, and out-coupler are highlighted in red, green, and blue boxes, respectively. The purple box in the geometric waveguide shows the out-coupled FOV, which is different from the incident FOV. The red, green, and blue arrows in the diffractive *k*-vector diagram indicate the grating vectors of the in-coupler, expander, and out-coupler, respectively

Now, writing in eLight, Ding et al. have comprehensively reviewed AR waveguide displays, providing timely information for the community^4^. The authors delve into the ambient contrast ratio (ACR) of an AR display, particularly in relation to the type of light engine responsible for creating the digital images, succinctly presented in Table 1 in ref. ^4^. The ratio of the perceived luminance in the on-state to that in the off-state determines the ACR. This perceived luminance combines the display’s luminance at the eye and the ambient luminance seen through the transparent waveguide.

A high-brightness light engine and an efficient waveguide are crucial to achieve a high ACR under bright ambient conditions. Additional metrics, including resolution density and frame rates, must also be elevated for image quality. These engines should ideally be compact, bright, and possess a high pixel count to facilitate more immersive displays. However, achieving a high pixel count in a small package necessitates shrinking already microscopic pixels. Table 1 in ref. ^4^ further provides a summarized overview of the prevailing research trends for each display engine type.

As outlined in Ding et al., waveguide combiners come with inherent challenges, encompassing limitations in FOV, eyebox, FOV uniformity and efficiency, and image sharpness^4^. Fundamental constraints, such as the maximum FOV constrained by the waveguide refractive index and the critical angle of TIR, directly influence the immersive potential of this architectural type and consequently impact its application space. Moreover, in-coupling efficiency is a limiting factor for system brightness since any light lost at the in-coupler cannot be recovered, presenting a bottleneck to overall efficiency^5^.

As highlighted in Ding et al., waveguide combiners can be broadly classified into geometric or diffractive types, depending on whether they rely on reflection and refraction (geometric) or diffraction (diffractive) to redirect and replicate the light^4,6^. Geometric waveguides employ embedded mirrors or prisms, as illustrated in Fig. 1, while diffractive waveguides use components like surface relief^7^, holographic^8^, or metasurface gratings^9^. Typically, waveguides adhere to one category of components, but there have been proposals for systems utilizing a hybrid approach with both geometric and diffractive components^10^.

The *k*-vector diagram, depicted in Fig. 1^6^, is a valuable tool for comprehending waveguide systems. A significant aspect of *k*-vector diagrams is that the TIR ring visually represents the maximum FOV that can be contained within the waveguide. Interactions within the waveguide shift the FOV around the diagram, but the FOV must stay within the ring to stay within the waveguide. Shifting inside the ring’s inner limit indicates a failure to meet the TIR condition, resulting in leakage. Shifting outside the ring implies that the FOV has become evanescent and is not physically realizable.

These *k*-vector diagrams are commonly employed for analyzing diffractive waveguides because, in *k*-space, diffractive interactions add linearly as vectors, as illustrated by the arrows in the diffractive *k*-vector diagram in Fig. 1. Following each interaction, the FOV (indicated by colored dots) shifts by the grating vector. The shape of the overall FOV and the relative position of each point in the FOV remain constant in *k*-space. *k*-vector diagrams play a crucial role in designing intricate pupil expansion schemes such as crossed gratings, which are capable of achieving expansion and out-coupling in a single region, as demonstrated by Ding et al.

Ding et al. also employ *k*-vector diagrams in the geometric case to facilitate a more insightful comparison between diffractive and geometric systems. In the geometric case, the FOV in the *kx*/ky projection shifts to the right after in-coupling compresses slightly, and flips, resulting in the left (blue) part of the FOV now appearing on the right. In contrast, in the diffractive case, the in-coupled FOV shifts to the right without compressing or flipping. The compression of the FOV demonstrates how the geometric waveguide combiners can support a larger FOV with the same refractive index as the diffractive waveguides. However, due to reflections flipping the FOV at in-coupling, expanding, and out-coupling, the FOV reflects about the $y=-x$ line for geometric waveguides. Analyzing both schemes in *k*-space facilitates a first-order analysis and understanding of light’s behavior in each system.

As the research and development of waveguide displays progresses, incorporating advanced metrics becomes crucial for comprehending the impact of new research and facilitating comparisons with prior work. Ding et al. provided common metrics applicable to all AR displays, including MTF, FOV, eyebox, uniformity, efficiency, form factor and weight, eye glow, rainbows, and ACR^4^.

Evolving research introduces novel metrics, such as presenting efficiency maps over the FOV rather than the average efficiency and uniformity as single values^5^. To fully capture the performance behavior of the display, it is important to understand trends over the FOV, which can be visualized in efficiency maps. Similar maps can be made over the eyebox to understand how efficiency for each field changes with eye position^11^. These maps can also present other information like image quality or dispersion. Mapping performance shows which fields or eyebox positions have the worst performance and need addressing.

To that end, summarizing waveguide performance as a single-value metric can be effectively done by reporting the minimum value over the FOV rather than the average or uniformity. For example, more efficient fields will appear brighter to the user than those with low efficiency. The more efficient fields can be dimmed at the display to present a uniform display to the user, but the dimmest field cannot be made brighter beyond the limits of the display. Thus, the minimum derived from the full field map can be presented as a single-value to summarize the limits of the display’s capabilities.

Analogous to a chain being only as strong as its weakest link, a display can be evaluated in terms of its most limiting features. Ongoing research into the effect of waveguide components on performance is also revealing how waveguide components set limits and tradeoffs for system performance. The future of waveguide research and development will benefit from a thorough understanding of how each component impacts performance and the ability to communicate these findings comprehensively and intuitively.
